# The association of lung function and St. George's respiratory questionnaire with exacerbations in COPD: a systematic literature review and regression analysis

**DOI:** 10.1186/s12931-016-0356-1

**Published:** 2016-04-16

**Authors:** Amber L. Martin, Jessica Marvel, Kyle Fahrbach, Sarah M. Cadarette, Teresa K. Wilcox, James F. Donohue

**Affiliations:** Evidera, 430 Bedford Street, Suite 300, 02420 Lexington, MA USA; Novartis Pharmaceuticals Corporation, East Hanover, NJ USA; University of North Carolina School of Medicine, Chapel Hill, NC USA

**Keywords:** COPD, Exacerbations, FEV_1_, SGRQ, Health resource utilization, Regression analysis

## Abstract

**Background:**

This study investigated the relationship between changes in lung function (as measured by forced expiratory volume in one second [FEV_1_]) and the St. George’s Respiratory Questionnaire (SGRQ) and economically significant outcomes of exacerbations and health resource utilization, with an aim to provide insight into whether the effects of COPD treatment on lung function and health status relate to a reduced risk for exacerbations.

**Methods:**

A systematic literature review was conducted in MEDLINE, Embase, and the Cochrane Central Register of Controlled Trials to identify randomized controlled trials of adult COPD patients published in English since 2002 in order to relate mean change in FEV_1_ and SGRQ total score to exacerbations and hospitalizations. These predictor/outcome pairs were analyzed using sample-size weighted regression analyses, which estimated a regression slope relating the two treatment effects, as well as a confidence interval and a test of statistical significance.

**Results:**

Sixty-seven trials were included in the analysis. Significant relationships were seen between: FEV_1_ and any exacerbation (time to first exacerbation or patients with at least one exacerbation, *p* = 0.001); between FEV_1_ and moderate-to-severe exacerbations (time to first exacerbation, patients with at least one exacerbation, or annualized rate, *p* = 0.045); between SGRQ score and any exacerbation (time to first exacerbation or patients with at least one exacerbation, *p* = 0.0002) and between SGRQ score and moderate-to-severe exacerbations (time to first exacerbation or patients with at least one exacerbation, *p* = 0.0279; annualized rate, *p* = 0.0024). Relationships between FEV_1_ or SGRQ score and annualized exacerbation rate for any exacerbation or hospitalized exacerbations were not significant.

**Conclusions:**

The regression analysis demonstrated a significant association between improvements in FEV_1_ and SGRQ score and lower risk for COPD exacerbations. Even in cases of non-significant relationships, results were in the expected direction with few exceptions. The results of this analysis offer health care providers and payers a broader picture of the relationship between exacerbations and mean change in FEV_1_ as well as SGRQ score, and will help inform clinical and formulary-making decisions while stimulating new research questions for future prospective studies.

## Background

Chronic obstructive pulmonary disease (COPD) is characterized by persistent airway obstruction related to chronic inflammatory responses in the lungs with symptoms including disabling dyspnea, fatigue, and persistent cough with excessive sputum. Exacerbations are characterized by a sustained acute worsening of respiratory symptoms beyond daily fluctuations, which leads to changes in medication use. Due to the disease symptoms, COPD patients often have a reduced capacity for physical activity and this may worsen potential systemic manifestations of the disease, such as cardiovascular and psychiatric comorbidities. The global prevalence of COPD is estimated to be 9.2 % [1] with variable estimates, ranging from 3.9 % [2] in the Netherlands to 20.9 % in the US, [3] when reported by country. Therefore, COPD presents a major clinical and humanistic burden, [4] despite the availability and use of standard treatments, which aim to relieve symptoms and slow disease progression [5].

This heavy disease toll inevitably focuses interest on how patients are treated and the extent to which medications produce meaningful benefits. Assessment of such value in clinical trials has traditionally relied on measures of lung function (such as forced expiratory volume in one second [FEV_1_]), symptom control, health status, and rates of exacerbation over a period of up to one year. Exacerbations are a particularly important marker, not least because they are a key driver of health resource use (HRU), such as emergency department visits, antibiotic use and hospitalization. Evidence of this includes the fact that an exacerbation can cost upwards of $7,000 each, depending on its severity and whether the patient is hospitalized [6]. Unsurprisingly, payers tend to focus on this outcome in their formulary considerations, with the expectation that decreased exacerbation rates will likely result in lower costs for their plan.

The clinical and economic importance of exacerbations in COPD invites questions about their inter-relationship with other well-established measures of treatment effect. These include, for example, persistent and/or uncontrolled disease symptoms and health status as measured by the St. George’s Respiratory Questionnaire [SGRQ] – which captures symptoms, impact on patient well-being, and activities of daily living. Additionally, clinically relevant improvements in lung function measures such as FEV_1_, are often required by regulators for certain drug approval processes. Of note, previous studies have looked at the link between FEV_1_ and SGRQ score [7, 8] but their relationship to longer-term outcomes, such as exacerbations and HRU, is not well-known and/or accepted, and this may account for why they have received comparatively less consideration from clinicians and payers.

Against this background, the current study aimed to investigate the relationship between changes in FEV_1_ and SGRQ score and economically significant outcomes of exacerbations and HRU, by conducting a systematic literature review (SLR) and regression analysis of relevant studies of pharmacological interventions for COPD. The results of this analysis will help the interpretation of clinical trial results and provide insights into whether or how the effects of COPD treatment seen in such studies relate to long-term clinical benefits.

## Methods

### Literature review

#### Search strategy

We systematically reviewed MEDLINE- (via PubMed), Embase-, and the Cochrane Central Register of Controlled Trials (CENTRAL) -indexed literature published from January 1, 2002 through October 1, 2014. The search algorithms used keywords for COPD paired with terms for the endpoints of interest--SGRQ, FEV_1_, exacerbations, and HRU. Limits included clinical trials on humans published in English.

#### Study selection

Following the literature search, all titles and abstracts identified from MEDLINE, Embase, and CENTRAL were manually reviewed against the inclusion and exclusion criteria using PICOS (Patient, Interventions, Comparisons, Outcomes, Study Design)-related elements. Studies were required to report on at least 20 adult COPD patients, to evaluate pharmacologic treatments labeled for or intended for use as treatment of COPD with any comparator treatment, to report mean change in either FEV_1_ or SGRQ score and either COPD exacerbations or any HRU endpoint, and to be a randomized controlled trial (RCT). A single investigator screened all abstracts identified through the searches, according to the specified inclusion and exclusion criteria. The full-text articles of accepted studies that passed abstract screening were retrieved for further review. Screening was conducted by a single investigator using the same inclusion and exclusion criteria that had been applied at the abstract level. All excluded studies were confirmed by a second, senior investigator and any discrepancies between the two investigators were resolved by involvement of a third investigator.

#### Data extraction process

The results of all accepted studies identified as part of the SLR were extracted by a single investigator trained in the critical assessment of evidence, with validation performed by a senior investigator. Trial quality and risk of bias were assessed during extraction for each included study using the Jadad quality score assessment.

### Statistical analysis

The analyses relating measures of FEV_1_ and SGRQ total score to exacerbations and HRU followed the meta-analyses methods outlined by Johnson et al. [9] Each trial supplied one or more pairs of data points on the treatment effects of interest. These predictor/outcome pairs from each of the studies were analyzed using sample-size weighted regression analyses, which estimated a regression slope relating the two treatment effects, as well as a confidence interval and a test of statistical significance. In general, the predictor was a relative treatment effect for change in SGRQ or trough FEV_1_, and the outcome was a log-relative-risk or log-rate for exacerbations. Pre-bronchodilator FEV_1_ was considered as equivalent to trough FEV_1_ for analysis, while post-bronchodilator measures and FEV_1_ that was unspecified were not included. Primary analyses were designed to avoid the use of an intercept in the regressions, but fit was superior with an intercept included.

For the analyses of patients experiencing at least one exacerbation, studies were included if they reported on exacerbations of all severities. For analyses of patients experiencing at least one moderate-to-severe exacerbation, studies were included if they reported on exacerbations that required antibiotics, oral corticosteroids (OCS), and/or hospitalization. Data on time to first exacerbation or the number of patients with at least one exacerbation were combined for analysis. COPD exacerbations reported as an adverse event were not included in analysis. All studies reporting data at timepoints ≥24 weeks were eligible for inclusion in the analyses. Separate analyses were conducted for all timepoints ≥24 weeks and ≥48 weeks.

## Results

### Literature review

**Fig. 1:**
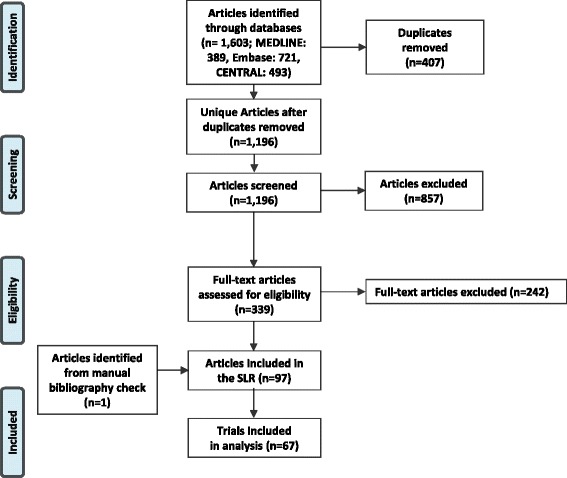
Study Attrition in the Systematic Literature Review

The literature review identified 67 trials reporting endpoints of interest at timepoints ≥24 weeks that were eligible for inclusion in the regression analysis. Fig. 1 outlines the overall search hits and study attrition during screening and analysis.

### Regression analysis

**Fig. 2:**
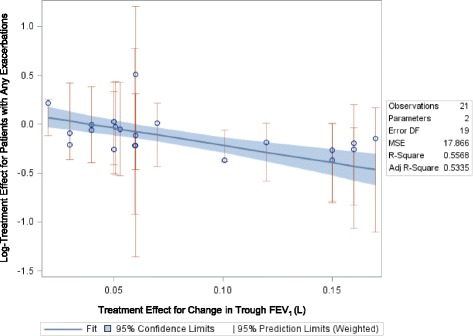
Relationship between Mean Change in Trough FEV_1_ and Relative Risk for Any Exacerbation

In the figures representing the analyses, each point in the plot represents a study comparison for two effects. For instance, the point in the middle of Fig. 2 is from Bateman et al. [10] and represents their findings in the comparison of tiotropium 5 mg (via the Respimat® inhaler) vs. placebo. In this example, the difference between the two treatments in trough FEV_1_ change was -0.10, and the hazard ratio (HR) for any exacerbation risk was 0.693 (for a log-HR of -0.37). Each study with two arms (one treatment comparison, e.g. treatment A vs. treatment B) and with sufficient data contributed one data point to the analysis; studies with three arms (two treatment comparisons, e.g. A vs. B and A vs. C) contributed two data points.

Any given slope can be interpreted by determining what difference between treatments in log-exacerbation risk one would expect given the difference in trough FEV_1_ change. The predicted log-relative-risk of exacerbation in studies like Bateman 2010 is:

$$ \ln \left(\mathrm{RRAnyExacerbation}\right) = \mathrm{Intercept} + \mathrm{Slope}\ *\ \mathrm{Difference}\ \mathrm{in}\ \mathrm{trough}\ {\mathrm{FEV}}_1\mathrm{change}. $$

Or

$$ \ln \left(\mathrm{RRAnyExacerbation}\right) = 0.14 - 3.56(0.10), = -0.22. $$

As exp (-0.22) = 0.80, we can predict that the relative risk of exacerbation in studies like Bateman 2010 will be 20 % lower for active treatment than for control. As noted above and in the plot, in Bateman 2010 the relative risk of any exacerbation was actually slightly lower than this value (0.693).

### Relationships with exacerbations at ≥48 weeks

#### Forced Expiratory Volume in One Second (trough FEV_1_)

##### Mean Change in Trough FEV_1_ and COPD Patients’ Risk for Any Exacerbation

The relationship between relative treatment effects on change in FEV_1_ and any exacerbation was of moderate strength and was statistically significant (slope: -3.56, *p* = 0.0001; Fig. 2) when defining the exacerbation outcome as time to first exacerbation or the number of patients with at least one exacerbation. No relationship was found (slope: 0.078, *p* = 0.9199) between treatment effects on FEV_1_ and annualized exacerbation rate. Figure 2 plots the relationship between the mean difference in trough FEV_1_ and relative risk for any exacerbation and Table 1 shows the raw trial data contributing to this analysis.

**Table 1:** Study Data for Trials Reporting Mean Change in Trough FEV_1_ and Patients Experiencing Any Exacerbation

Author, Year	Treatment	Time point (weeks)	N Randomized	Definition of exacerbation	Annual exacerbation rate	N with any exacerbation	Comparison data for Time to first exacerbation (Hazard ratio)	Mean change in Trough FEV_1_ (L)	Comparison data for Trough FEV_1_ (treatment difference)
Bateman, 2010 [10]	Tiotropium 5 ug	48	1989	B+	0.12	685	Tio5 vs. Placebo: 0.69	0.119	--
	Placebo	48	2002		0.15	842		0.018	--
Calverley, 2010 [12]	Beclomethasone/formoterol pMDI 400/24 μg	48	237	NR	0.074	64	--	0.077	B/F pMDI vs. F-DPI: 0.051
	Budesonide/formoterol DPI)800/24 μg	48	242		0.033	64	--	0.08	B/F dry vs. F-DPI: 0.053
	Formoterol DPI 12 μg	48	239		0.04	66	--	0.026	--
Chapman, 2011 [13]	Indacaterol, 150 μg	52	420	A	--	--	Ind150 vs. Placebo: 0.82	0.12	--
	Indacaterol, 300 μg	52	418		--	--	Ind300 vs. Placebo: 0.86	0.13	--
	Placebo	52	425		--	--		-0.04	--
Dahl, 2010 [14]	Indacaterol 300 μg	52	437	A	--	--	Inda300 vs. Placebo: 0.77	--	Inda300 vs. Placebo: 0.16
	Indacaterol 600 μg	52	428		--	--	Inda600 vs. Placebo: 0.69	--	Inda600 vs. Placebo: 0.15
	Formoterol	52	435		--	--	F vs. Placebo: 0.77	--	F vs. Placebo: 0.05
	Placebo	52	432		--	--		--	--
Decramer, 2013 [15]	Tiotropium bromide 18 μg	26	1721	C	--	--	Tio18 vs. Inda150: 0.81	--	Tio18 vs. Inda150: 0.02
	Indacaterol maleate 150 μg once-daily	26	1723		--		--	--	--
	Tiotropium bromide 18 μg	52	1721		0.07	547	--	0.092	--
	Indacaterol maleate 150 μg once-daily	52	1723		0.1	619	--	0.073	--
Dusser, 2006 [16]	Tiotropium 18 μg once daily	48	500	C	--	248	--	--	--
	Placebo	48	510		--	305	--	--	Tio18 vs. Placebo: 0.12
Ferguson, 2008 [17]	Fluticasone propionate/salmeterol (FSC) 250/50	52	394	C	--	343	--	-0.012	--
	Salmeterol 50 μg	52	388		--	335	--	-0.082	--
van Grunsven, 2003 [18]	Fluticasone propionate (Flixotides) 250 μg bid	103	24	D	--	5	--	-0.12	F250 vs. Placebo: 0.06
	Placebo bid	103	24		--	3	--	-0.17	--
Vincken, 2002 [19]	Tiotropium 18 μg qd in the morning	52	356	B	--	125	--	0.12	--
	Ipratropium 40 μg qid	52	179		--	82	--	-0.03	--
Wouters, 2005 [20]	Salmeterol/fluticasone (3 month run in period of salmeterol 50 μg and fluticasone 500 μg bid)	52	189	E	--	115	--	-0.04	S/F vs. S: 0.05
	Salmeterol (3 month run in period of salmeterol 50 μg and fluticasone 500 μg bid)	52	184		--	109	--	-0.1	--
Zhou, 2006 [21]	Theophylline	52	57	C	--	26	--	0.0063	--
	Placebo	52	53		--	30	--	-0.0533	--
Dransfield, 2013 [22]	Vilanterol 25 μg	52	409	A	--	203	--	-0.04	--
	Fluticasone furoate 50 μg + Vilanterol 25 μg	52	408		--	190	--	0	--
	Fluticasone furoate 100 μg + Vilanterol 25 μg	52	403		--	161	--	0.02	--
	Fluticasone furoate 200 μg + Vilanterol 25 μg	52	402		--	178	--	0.02	--
	Vilanterol 25 μg	52	409		--	197	--	-0.02	--
	Fluticasone furoate 50 μg + Vilanterol 25 μg	52	412		--	198	--	0.02	--
	Fluticasone furoate 100 μg + Vilanterol 25 μg	52	403		--	177	--	0.01	--
	Fluticasone furoate 200 μg + Vilanterol 25 μg	52	409		--	160	--	0.01	--

Exacerbation Definitions:

A:Symptom deterioration requiring antibiotics, systemic corticosteroids, and/or hospitalization

B:A complex of respiratory events lasting ≥3 days

B+:A complex of respiratory events lasting ≥3 days requiring treatment

C:Worsening of at least two symptoms for at least two days

D:Having two of the following three symptoms: increased cough, wheezing and/or dyspnea; change in sputum color; use of bronchodilator rescue medication

E:If a patient has in ≥2 consecutive days used ≥3 extra inhalations of salbutamol per 24 hours above their reference rescue value

-- = Not Reported

##### Mean Change in Trough FEV_1_ and COPD Patients’ Risk for Moderate-to-Severe Exacerbations

**Fig. 3:**
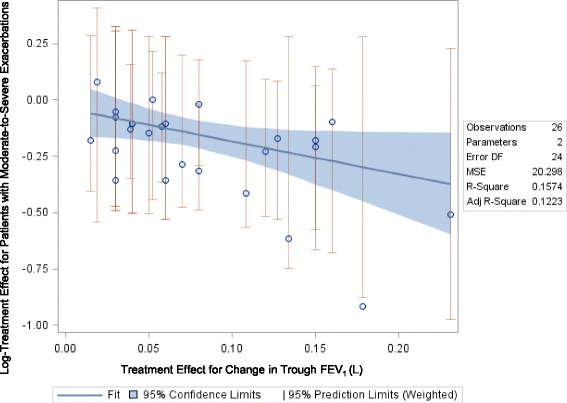
Relationship between Mean Change in Trough FEV_1_ and Risk for a Moderate-to-Severe Exacerbation

The relationship between relative treatment effects on change in FEV_1_ and moderate-to-severe exacerbations was of moderate strength and was statistically significant (slope: -1.46, *p* = 0.045; Fig. 3) when defining the exacerbation outcome either as time to first exacerbation, the number of patients with at least one exacerbation, or as annualized exacerbation rates. Figure 3 shows the relationship between the mean difference in trough FEV_1_ and the relative risk for a moderate-to-severe exacerbation. Table 2 shows the raw trial data contributing to this analysis.

**Table 2:** Study Data for Trials Reporting Mean change in FEV_1_ and Patients Experiencing Moderate-to-Severe COPD Exacerbation

Author, Year	Treatment	Time point (weeks)	N Randomized	Annual exacerbation rate (M-S)	N with M-S exacerbation	Comparison data for Time to first exacerbation (Hazard ratio)	Mean change in Trough FEV_1_ (L)	Comparison data for Trough FEV_1_ (treatment difference)
Anzueto, 2009 [23]	Fluticasone propionate/salmeterol 250 mcg/50 mcg bid	52	394	1.1	208	FP250 + S50 vs. S50: 0.73	-0.017	--
	Salmeterol 50 mcg bid	52	403	1.59	234	--	-0.097	--
Bateman, 2010 [10]	Tiotropium 5 μg orally inhaled once daily	48	670	0.93	249	--	0.08	Tio5 vs. Placebo: 0.127
	Tiotropium 10 μg orally inhaled once daily	48	667	1.02	246	--	0.11	Tio10 vs. Placebo: 0.150
	Placebo	48	653	1.91	288	--	-0.04	
Dahl, 2010 [14]	Indacaterol 300 μg	52	437	0.6	133	--	--	Inda300 vs. Placebo: 0.16
	Indacaterol 600 μg	52	428	0.57	116	--	--	Inda600 vs. Placebo: 0.15
	Formoterol	52	435	0.56	126	--	--	F vs. Placebo: 0.05
	Placebo	52	432	0.74	145	--	--	
Donohue, 2014 [24]	UMEC/VI 125/25 mcg	52	226	--	30	UMEC/VI vs. Placebo: 0.6	0.18	UMEC/VI vs. Placebo: 0.231
	UMEC 125 mcg	52	227	--	34	UMEC vs. Placebo: 0.4	0.13	UMEC vs. Placebo: 0.178
	Placebo	52	109	--	26	--	-0.05	--
Ferguson, 2008 [17]	Fluticasone propionate/salmeterol (FSC) 250/50	52	394	1.06	211	FP/S vs. S: 0.75	-0.012	--
	Salmeterol 50 μg	52	388	1.53	230	--	-0.082	--
Kerwin, 2012 [25]	NVA237 50 μg qd	52	529	0.54		NVA vs. Placebo: 0.66	0.112	NVA vs. Placebo: 0.108
	Tiotropium 18 μg qd	52	268	--	--	NVA vs. Tio: 1.1	0.092	NVA vs. Tio: 0.019
	Placebo	52	269	0.8	--	--	-0.097	
Sharafkhaneh, 2012 [26]	Budesonide/formoterol pMDI 160/4.5 μg x 2 inhalations bid (320/9 μg)	52	407	0.867	169	--	0.07	--
	Budesonide/formoterol pMDI 80/4.5 μg x 2 inhalations bid (160/9 μg)	52	408	0.952	173	--	0.07	--
	Formoterol DPI 4.5 μg x 2 inhalations bid (9 μg)	52	404	1.171	182	--	0.04	--
Tang, 2013 [27]	Tiotropium 5 μg (2 x 2.5 μg/puff)	48	167	--	58	Tio5 vs. Placebo: 0.54	--	Tio5 vs. Placebo: 0.134
	Placebo (2 puffs)	48	171	--	83	--	--	--
Tashkin, 2008 [11]	Tiotropium 18 μg once daily; followed by 40 μg of ipratropium four times daily for 30 days after 4 years of treatment.	206	2987	--	2001	--	0.03	--
	Placebo once daily; followed by 40 μg of ipratropium four times daily for 30 days after 4 years of treatment.	206	3006	--	2049	--	-0.05	--
Calverley, 2009 [28]	Roflumilast 500 mcg once per day	52	765	1.08	344	ROLF500 vs. Placebo (Trial 1): 0.88	0.046	ROLF500 vs. Placebo (Trial 1): 0.039
	Placebo	52	758	1.27	389	--	0.008	--
	Roflumilast 500 mcg once per day	52	772	1.21	373	ROLF500 vs. Placebo (Trial 2): 0.89	0.033	ROLF500 vs. Placebo (Trial 2): 0.058
	Placebo	52	796	1.49	432	--	-0.025	--
Dransfield, 2013 [22]	Vilanterol 25 μg	52	409	1.05	--	FF200 + V vs. V: 0.9	-0.04	--
	Fluticasone furoate 50 μg + Vilanterol 25 μg	52	408	0.92	--	FF100 + V vs. V: 0.7	0	--
	Fluticasone furoate 100 μg + Vilanterol 25 μg	52	403	0.7	--	FF50 + V vs. V: 0.9	0.02	--
	Fluticasone furoate 200 μg + Vilanterol 25 μg	52	402	0.9	--	--	0.02	--
	Vilanterol 25 μg	52	409	1.14	--	FF200 + V vs. V: 0.7	-0.02	--
	Fluticasone furoate 50 μg + Vilanterol 25 μg	52	412	0.92	--	FF100 + V vs. V: 0.8	0.02	--
	Fluticasone furoate 100 μg + Vilanterol 25 μg	52	403	0.9	--	FF50 + V vs. V: 0.9	0.01	--
	Fluticasone furoate 200 μg + Vilanterol 25 μg	52	409	0.79	--	--	0.01	--
Jones, 2011 [29]	Aclidinium 200 μg	52	627		167	Aclid200 vs. Placebo (Trial 1): 0.00	-0.013	
	Placebo	52	216	0.46	55	--	-0.065	--
	Aclidinium 200 μg	52	600		199	--	-0.009	--
	Placebo	52	204	0.8	81	--	-0.024	--

M-S = moderate-to-severe

-- = Not reported

#### St. George’s respiratory questionnaire

##### Mean Change in SGRQ Total Score and COPD Patients’ Risk for Any Exacerbations

**Fig. 4:**
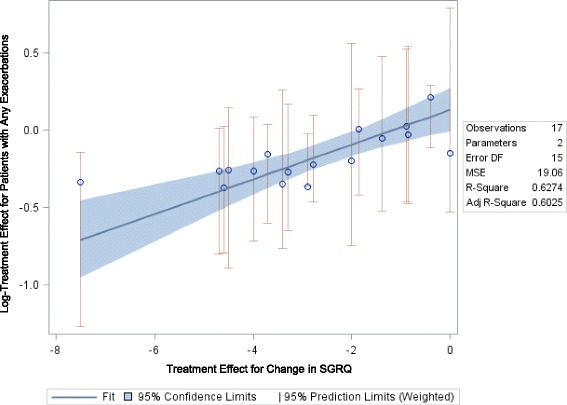
Relationship between Mean Change in SGRQ Total Score and Risk for Any Exacerbation

The relationship between relative treatment effects for change in SGRQ score and any exacerbation was of moderate strength (slope: 0.112, *p* = 0.0002; Fig. 4) and was statistically significant when defining the exacerbation outcome as time to first-exacerbation or the number of patients with at least one exacerbation. The relationship was weaker and not statistically significant (slope: 0.014, *p* = 0.2825) when examining annualized exacerbation rates. Figure 4 shows the relationship between the mean difference in SGRQ score and relative risk for any exacerbation and Table 3 shows the raw trial data contributing to this analysis.

**Table 3:** Study Data for Trials Reporting Mean change in SGRQ Total Score and Patients Experiencing Any COPD Exacerbation

Author, year	Treatment	Time point (weeks)	N Randomized	Definition of exacerbation	Annual exacerbation rate (any)	N with any exacerbation	Comparison data for Time to first exacerbation (Hazard ratio)	Mean change in SGRQ Total Score	Comparison data for SGRQ (treatment difference)
Bateman, 2010 [10]	Tiotropium 5 μg	48	1989	B+	0.69	685	Tio vs. placebo: 0.93	-4.7	Tio5 vs. placebo: -2.9
	Placebo	48	2002		0.87	842		-1.8	--
Calverley, 2003 [30]	Budesonide/formoterol 320/9 mg (bid)	52	254	A	1.38	--	B + F vs. B: 0.77	--	B + F vs. B: -4.5
	Budesonide 400 mg (bid)	52	257		1.6	--	B + F vs. F: 0.71	--	B + F vs. F: -3.4
	Formoterol 9 mg (bid)	52	255		1.85	--	B + F vs. Placebo: 0.72	--	B + F vs. Placebo: -7.5
	Placebo	52	256		1.8	--	--	--	--
Calverley, 2010 [12]	Beclomethasone/formoterol pMDI 400/24 μg	48	237	NR	0.414	64	--	-3.75	--
	Budesonide/formoterol DPI 800/24 μg	48	242		0.423	64	--	-4.28	--
	Formoterol DPI 12 μg	48	239		0.431	66	--	-2.9	--
Casaburi, 2002 [31]	Tiotropium 18 μg	52	550	B	0.76	198	--	-3.2	--
	Placebo	52	371		0.95	156	--	0.5	--
Chapman, 2011 [13]	Indacaterol, 150 μg	52	420	A	--	--	Ind150 vs. Placebo: 0.82	-7.5	--
	Indacaterol, 300 μg	52	418		--	--	Ind300 vs. Placebo: 0.86	-5.5	--
	Placebo	52	425		--	--	--	-5.5	--
Dahl, 2010 [14]	Indacaterol 300 μg	52	437	A	--	--	Inda300 vs. Placebo: 0.77	-6.5	Inda300 vs. Placebo: -4.7
	Indacaterol 600 μg	52	428		--	--	Inda600 vs. Placebo: 0.69	-7.2	Inda600 vs. Placebo: -4.6
	Formoterol	52	435		--	--	F vs. Placebo: 0.77	-7	F vs. Placebo: -4
	Placebo	52	432		--	--	--	-1.7	--
Decramer, 2013 [15]	Tiotropium bromide 18 μg	26	1721	C	--	--	--	-5.2	--
	Indacaterol maleate 150 μg once-daily	26	1723		--	--	--	-4.5	--
	Tiotropium bromide 18 μg	52	1721		0.61	547	--	-4.9	--
	Indacaterol maleate 150 μg once-daily	52	1723		0.79	619	--	-4.5	--
Ferguson, 2008 [17]	Fluticasone propionate/salmeterol (FSC) 250/50	52	394	C	4.82	343	--	-3.49	FP/S vs. S: -1.86
	Salmeterol 50 μg	52	388		5.78	335	--	-1.86	--
Vincken, 2002 [19]	Tiotropium 18 μg qd in the morning	52	356	B	0.73	125	--	-3.74	Tio18 vs. Ipra40: -3.3
	Ipratropium 40 μg qid	52	179		0.96	82	--	-0.44	--
Wedzicha, 2014 [32]	beclomethasone dipropionate/formoterol fumarate (BDP/FOR) 100/6 μg, 2 inhalations BID	48	602	F	0.8	264	BDP + F vs. F: 0.8	-3.55	BDP/F vs. F: -2.78
	Formoterol fumarate (FOR) 12 μg, 1 inhalation BID	48	597		1.12	294	--	-0.77	--
Wouters, 2005 [20]	Salmeterol/fluticasone (3 month run in period of salmeterol 50 μg and fluticasone 500 μg bid)	52	189	E	--	115	--	2.4	S/F vs. S: -0.89
	Salmeterol (3 month run in period of salmeterol 50 μg and fluticasone 500 μg bid)	52	184		--	109	--	3.2	--

Exacerbation Definitions:

A:Symptom deterioration requiring antibiotics, systemic corticosteroids, and/or hospitalization

B:A complex of respiratory events lasting ≥3 days

B+:A complex of respiratory events lasting ≥3 days requiring treatment

C:Worsening of at least two symptoms for at least two days

E:If a patient has in ≥2 consecutive days used ≥3 extra inhalations of salbutamol per 24 hours above their reference rescue value

F:An acute event characterized by a worsening of the patient's respiratory symptoms that is beyond normal day-to-day variations and leads to a change in medication

-- = Not reported

##### Mean Change in SGRQ Total Score and COPD Patients’ Risk for Moderate-to-severe Exacerbations

**Fig. 5:**
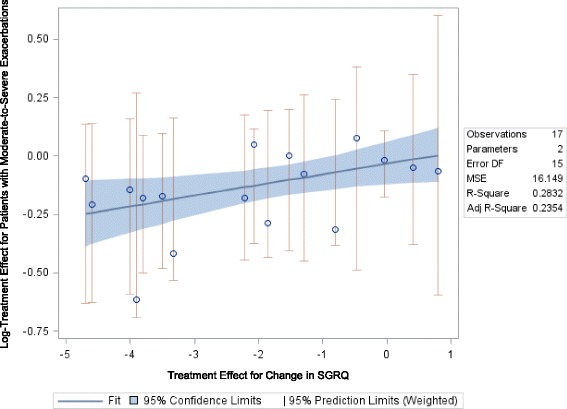
Relationship between Mean Change in SGRQ Total Score and Risk for a Moderate-to-severe Exacerbation

The relationship between relative treatment effects for change in SGRQ score and a moderate-to-severe exacerbation was of moderate strength and was statistically significant when defining the exacerbation outcome as either the number of patients with at least one exacerbation (slope: 0.046, *p* = 0.0279, Fig. 5) or as an annualized exacerbation rate (slope: 0.056, *p* = 0.0024, figure not shown). Figure 5 shows the relationship between the mean difference in SGRQ score and the relative risk for a moderate-to-severe exacerbation and Table 4 shows the raw trial data contributing to this analysis.

**Table 4:** Study Data for Trials Reporting Mean change in SGRQ Total Score and Patients Experiencing Moderate-to-severe COPD Exacerbation

Author, Year	Treatment	Time point (weeks)	N Randomized	Annual exacerbation rate (M-S)	N with M-S exacerbation	Comparison data for Time to first exacerbation (Hazard ratio)	Mean change in SGRQ Total Score	Comparison data for SGRQ (treatment difference)
Anzueto, 2009 [23]	Fluticasone propionate/salmeterol 250 mcg/50 μg bid	52	394	1.1	208	FP250 + S50 vs. S50: 0.73	2.49	FP250 + S50 vs. S50: -0.81
	Salmeterol 50 μg bid	52	403	1.59	234	--	3.28	--
Bateman, 2010 [20]	Tiotropium 5 μg orally inhaled once daily	48	670	0.93	249	--	-5.1	Tio5 vs. Placebo: -3.5
	Tiotropium 10 μg orally inhaled once daily	48	667	1.02	246	--	-5.5	Tio10 vs. Placebo: -3.8
	Placebo	48	653	1.91	288	--	-1.6	--
Dahl, 2010 [14]	Indacaterol 300 μg	52	437	0.6	133	--	-6.5	Inda300 vs. Placebo: -4.7
	Indacaterol 600 μg	52	428	0.57	116	--	-7.2	Inda600 vs. Placebo: -4.6
	Formoterol	52	435	0.56	126	--	-7	F vs. Placebo: -4
	Placebo	52	432	0.74	145	--	-1.7	--
Ferguson, 2008 [17]	Fluticasone propionate/salmeterol (FSC) 250/50	52	394	1.06	211	FP + S vs. S: 0.75	-3.49	FP/S vs. S: -1.86
	Salmeterol 50 μg	52	388	1.53	230	--	-1.86	--
Hagedorn, 2013 [33]	Salmeterol xinafoate/fluticasone propionate via a single inhaler (SFC)	52	108	0.81	42	--	-1.8	--
	Salmeterol xinafoate/fluticasone propionate via separate inhalers (Sal/FP)	52	106	0.98	44	--	-2.6	--
Kerwin, 2012 [25]	NVA237 50 μg qd	52	529	0.54	--	NVA vs. Placebo: 0.66	--	NVA vs. Placebo: -3.32
	Tiotropium 18 μg qd	52	268		--	NVA vs. Tio: 1.1	--	NVA vs. Tio: -0.48
	Placebo	52	269	0.8	--	--	--	--
Sharafkhaneh, 2012 [26]	Budesonide/formoterol pMDI 160/4.5 μg x 2 inhalations bid (320/9 μg)	52	407	0.867	169	--	-7.2	--
	Budesonide/formoterol pMDI 80/4.5 μg x 2 inhalations bid (160/9 μg)	52	408	0.952	173	--	-5.5	--
	Formoterol DPI 4.5 μg x 2 inhalations bid (9 μg)	52	404	1.171	182	--	-5.9	--
Tang, 2013 [27]	Tiotropium 5 μg (2 x 2.5 μg/puff)	48	167	--	58	Tio5 vs. Placebo: 0.54	-7.1	Tio5 vs. Placebo: -3.9
	Placebo (2 puffs)	48	171	--	83	--	-3.3	--
Tashkin, 2008 [11]	Tiotropium 18 μg once daily; followed by 40 μg of ipratropium four times daily for 30 days after 4 years of treatment.	206	2987	--	2001	--	-1.25	--
	Placebo once daily; followed by 40 μg of ipratropium four times daily for 30 days after 4 years of treatment.	206	3006	--	2049	--	-1.21	--
Wedzicha, 2008 [34]	Salmeterol 50 μg + fluticasone propionate 500 μg bid	104	658	--	408	--	-1.7	--
	Tiotropium bromide 18 μg once daily	104	665	--	392	--	0.37	S + F vs. Tio18: -2.07
Jones, 2011 [29]	Aclidinium 200 μg	52	627	--	167	Aclid200 vs. Placebo (trial 1): 1.00	--	Aclid200 vs. Placebo (trial 1): -1.53
	Placebo	52	216	--	55	--	--	--
	Aclidinium 200 μg	52	600	--	199	--	--	Aclid200 vs. Placebo (trial 2): -2.21
	Placebo	52	204	--	81	--	--	--

M-S = moderate-to-severe

-- = Not reported

### Relationship between FEV_1_ and SGRQ and Hospitalized COPD Exacerbations

There were insufficient data to analyze association with all-cause hospitalizations, and the annualized and patient-level data were combined for the analysis of hospitalizations due to exacerbations. Additionally, relative effects for the number of patients with an exacerbation were combined with annualized exacerbation rates to facilitate analyses.

#### FEV_1_ and SGRQ

**Fig. 6:**
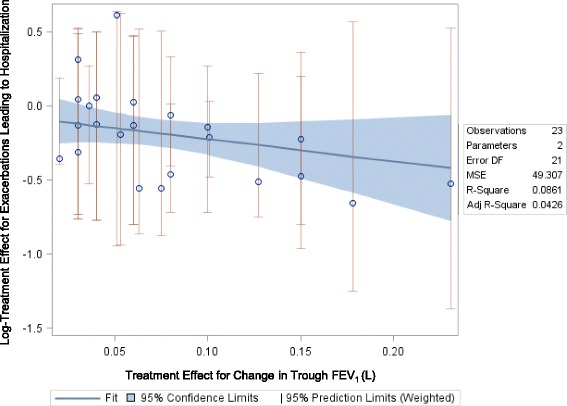
Relationship between Mean Change in FEV_1_ and Risk for Hospitalization

**Fig. 7:**
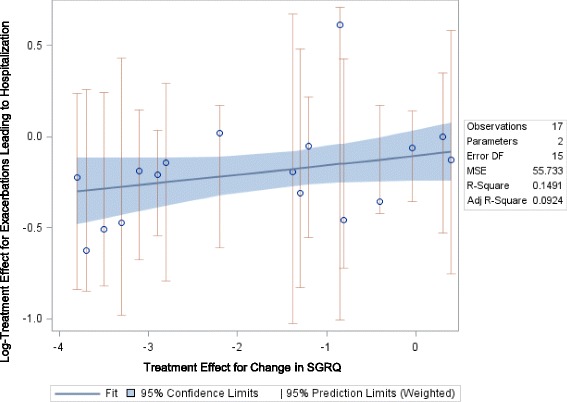
Relationship between Mean Change in SGRQ and Risk for Hospitalization

For both SGRQ score and FEV_1,_ the plots indicate a somewhat weaker relationship with exacerbations resulting in hospitalization (compared to the findings for exacerbations overall). Results were not statistically significant (FEV_1_ slope: -1.49, *p*-value = 0.174 [Fig. 6]; SGRQ slope: 0.0518, *p* = 0.126 [Fig. 7]) for either relationship.

### Impact of including All timepoints >24 weeks

Expanding the data set from outcomes reported at >48 weeks to include outcomes reported at >24 weeks showed similar directionality but weaker results compared with the long-term analysis data of both SGRQ score and FEV_1_ (data not shown).

## Discussion

Our systematic literature review and regression analysis demonstrated that beneficial mean change in either FEV_1_ or SGRQ total score was associated with a lower risk for exacerbations. Specifically, it showed that in randomized trials of COPD drug treatments lasting ≥48 weeks, there was generally a relationship between relative efficacy in improving FEV_1_ and SGRQ total score and relative efficacy for lowering exacerbation risk. The majority of analyses showed the same trend towards a relationship between positive changes in FEV_1_ and SGRQ score and exacerbation risk, even though results did not always reach statistical significance. Of note, there was no relationship shown between mean change in FEV_1_ and annualized exacerbation rate, despite this relationship being moderate and statistically significant when the risk of experiencing at least one exacerbation in patients was analyzed. The mean change in SGRQ total score was not significantly related to the rate of exacerbations across all severities but had a moderate, statistically significant relationship with the rate of moderate-to-severe exacerbations. The relationship between FEV_1_ and SGRQ score and hospitalizations was less clear, and further research is needed in this area.

To our knowledge, the literature review and regression analysis we conducted is the first such study to evaluate the inter-relationship that health status and lung function have with exacerbation risk. It provides a more rigorous examination of a relationship between laboratory values and exacerbations than has been done in the past, as, unlike former studies, it correlates relative treatment effects instead of absolute ones, thus lowering the possibility of ecological bias. However, as this analysis used only aggregated patient data from published trials, we cannot assume that any statistical association observed between arm-level variables may be translated to patient-level associations. Therefore, our findings cannot be used to predict any outcome at the patient-level. Additionally, our analysis may be limited by the available data for the surrogate measures given the trials reported FEV_1_ in several different ways. Since our analysis was limited to trough or prebronchodilator FEV_1_ data, analysis using other measures of FEV_1_ could yield different results. Similarly, regarding exacerbation severity, we categorized exacerbations based on the definitions reported by study authors using a standardized approach as defined in our methods section. However, in some cases definitions were not reported so we relied on author-defined groupings of any or moderate-to-severe exacerbations.

Our research may have important implications for regulatory assessment of drugs intended to help reduce the risk of exacerbations in COPD and, in particular, the evidence considered in such deliberations. Currently, to gain marketing approval for this indication, such treatments have to be tested in long-term, parallel trials, which represent a logistic and economic burden on the sponsoring organization. Because of this, few trials of COPD drugs are powered to identify a significant difference in the reduced risk of exacerbations. It is for this reason that to date very few drugs have been approved for reducing exacerbations on the basis of prospective 1–2 year parallel trials, usually in patients with history of acute exacerbations in the prior year. Our study suggests changes in FEV_1_ and SGRQ might serve as reliable surrogate markers of patients’ likelihood of experiencing an exacerbation. If so, these measures could allow future trials to be shorter and more manageable while still offering key insights into treatments’ longer-term efficacy. Since exacerbations can be costly to health plans, payers should consider the effect of medications on these surrogate markers, even when long-term RCTs cannot be carried out. Also, confirmation of our results would broaden the application of data already available from published shorter-term studies. This is especially important since the trials used to inform regulatory approval were powered on each specific drug’s expected effect on the acute exacerbation rate and all but one [11] were small and had very selective entry criteria. This contrasts with the trials contributing data for our review and analysis, since these were broader and more inclusive (e.g. with regards to disease duration and reversibility, comorbidities, interventions, and concomitant therapies) and collectively more representative of the general COPD population seen in everyday clinical practice. Therefore, these collated data sources potentially allow more generalizable conclusions to be drawn regarding whether or how standard short-term endpoints assessed in trials relate to effects on exacerbations.

## Conclusions

In conclusion, this study demonstrates a significant association between improvements in FEV_1_ and SGRQ total score and lower risk for COPD exacerbations. We believe that the results of our study offer providers and payers a more informed picture of the inter-relationship between exacerbations and both FEV_1_ and SGRQ score, which will aid clinical and formulary decisions while stimulating research questions for future prospective studies.

## Abbreviations

COPD: chronic obstructive pulmonary disease
FEV_1_: forced expiratory volume in one second
HR: hazard ratio
HRU: health resource use
OCS: oral corticosteroids
PICOS: Patient, Interventions, Comparisons, Outcomes, Study Design
RCT: randomized controlled trial
SGRQ: St. George’s Respiratory Questionnaire
SLR: systematic literature review
